# Common Femoral Artery Thrombosis Post Hip Hemiarthroplasty: A Case Report

**DOI:** 10.7759/cureus.87263

**Published:** 2025-07-04

**Authors:** Yasir A Al-Humairi, Mohammed H Al-Haideri, Ali H Ismaeil

**Affiliations:** 1 Orthopaedics and Trauma, Rashid Hospital, Dubai, ARE; 2 Orthopaedics and Trauma, Dubai Health, Rashid Hospital, Dubai, ARE; 3 Trauma and Orthopaedic Surgery, Rashid Hospital, Dubai, ARE; 4 Trauma and Orthopaedic Surgery Residency Program, Dubai Health, Dubai, ARE; 5 Clinical Instruction, Mohammad Bin Rashid University, Dubai, ARE

**Keywords:** angiography, arthroplasty, common femoral artery occlusion, hemiarthroplasty, hip, thrombosis

## Abstract

Femur neck fractures are among the most common presentations seen in trauma centers. Surgical fixation of the fracture versus hip joint arthroplasty is the standard modality of treatment. The appropriate treatment modality is decided based on the patient’s fracture pattern, activity level prior to the injury, fitness for surgery, and availability of resources. Total or hemi hip arthroplasties are effective and well-optimized procedures. However, they do not come without the risk of complications, some of which can cause significant morbidity to the patient. We present a case of a vascular complication in a femur neck fracture, treated through a hemi hip arthroplasty. Our patient is a 67-year-old woman with a known history of hypertension and a long-standing history of heavy smoking. Admitted as a case of a basicervical femur neck fracture sustained secondary to a fall from a standing height, she underwent an uneventful hip hemiarthroplasty. However, her distal limb pulses were not palpable in the recovery area postoperatively and were not detectable on a Doppler ultrasound. CT angiography revealed an arterial thrombus and intimal tear in the common femoral artery. An arterial thrombectomy and an intimal repair were performed, and good limb perfusion was restored. Arterial injuries during joint replacement surgery are rare but serious complications. Prompt investigations in the form of a Doppler ultrasound of the distal pulses and CT angiography with urgent vascular surgery consultation, if needed, are crucial. If detected and treated early, limb perfusion can be restored. However, late detection could lead to drastic outcomes, which can include loss of limb and loss of function. The risk of perioperative vascular complications can be minimized intraoperatively through proper handling of surgical instruments and careful dissection and traction of limbs. Pre- and postoperative physical examinations, as well as having a high index of suspicion for such injuries, are important for early detection and better outcomes.

## Introduction

Femoral neck fractures are among the most common fractures sustained by the elderly population. These fractures typically occur secondary to low-energy falls and minor trauma. Surgical management in the form of hemiarthroplasty or total hip arthroplasty is the mainstay of treatment for this type of injury. The main aim of surgical intervention is to restore the patient’s pre-injury level of activity. Vascular complications following hemiarthroplasty or total hip arthroplasty are rare and can be found in a few reported cases [1-6]. The incidence of such complications is reported in the existing literature to be between 0.2% and 0.3% [7]. Spontaneous arterial occlusion through thrombosis, vascular perforation with surgical instruments, and vascular tears while manipulating implants have all been described [8]. This paper reports a case of femur neck fracture managed through hemiarthroplasty, where the patient suffered a severe vascular complication.

## Case presentation

**Figure 1:**
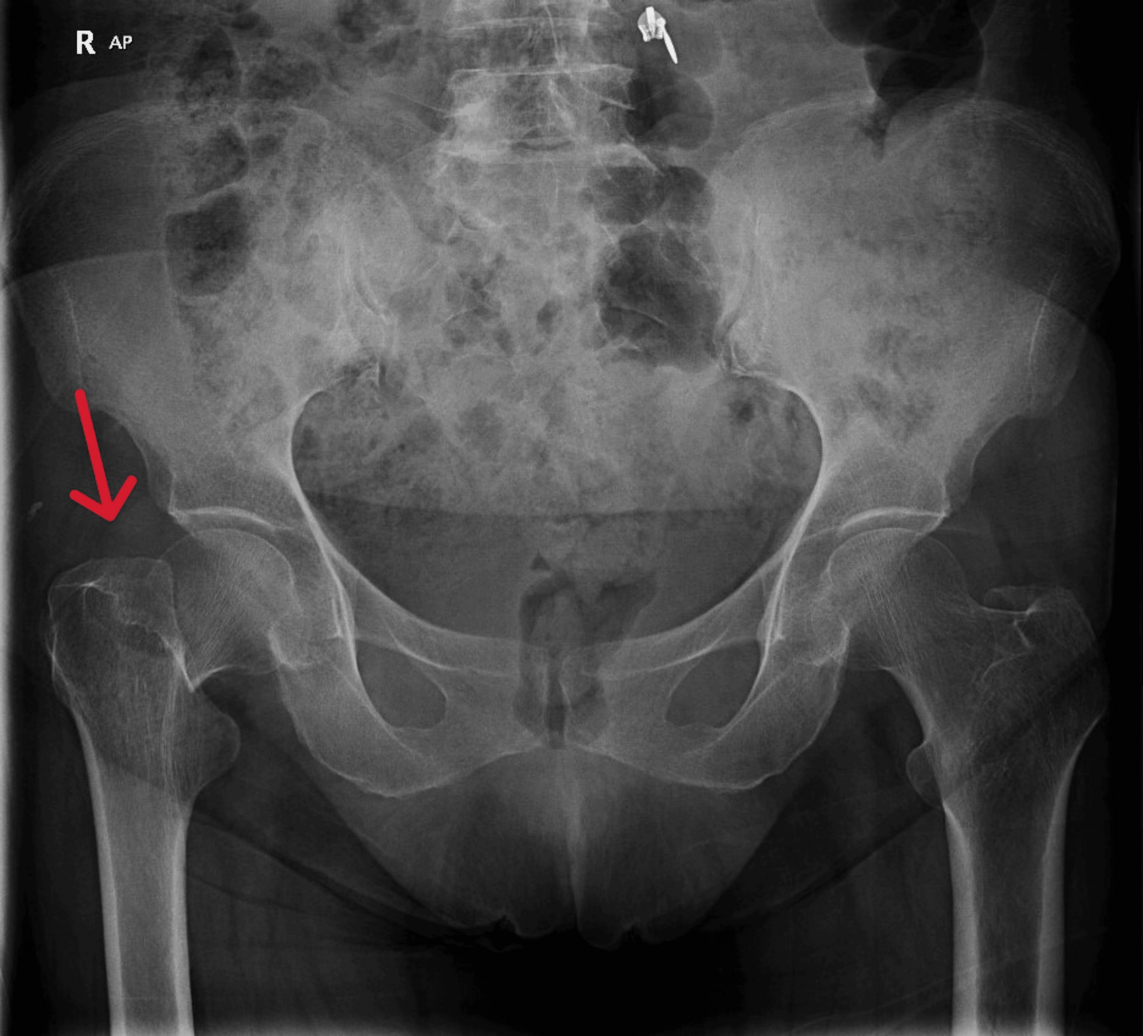
AP view of the hip and pelvis showing a basicervical femoral neck fracture in the right femur (red arrow) AP: Anteroposterior

We present a case of a 67-year-old Middle Eastern lady with a known history of controlled primary hypertension and a chronic history of smoking. She had no other pertinent past medical or surgical history. She was admitted to our level 1 trauma facility after sustaining an injury to her right hip. She described a history of a fall from a standing height while getting out of her motor vehicle. She sustained an isolated closed fracture of her right femur neck. The diagnosis was confirmed on imaging and classified as a basicervical neck-of-femur fracture (Figure 1).

**Figure 2:**
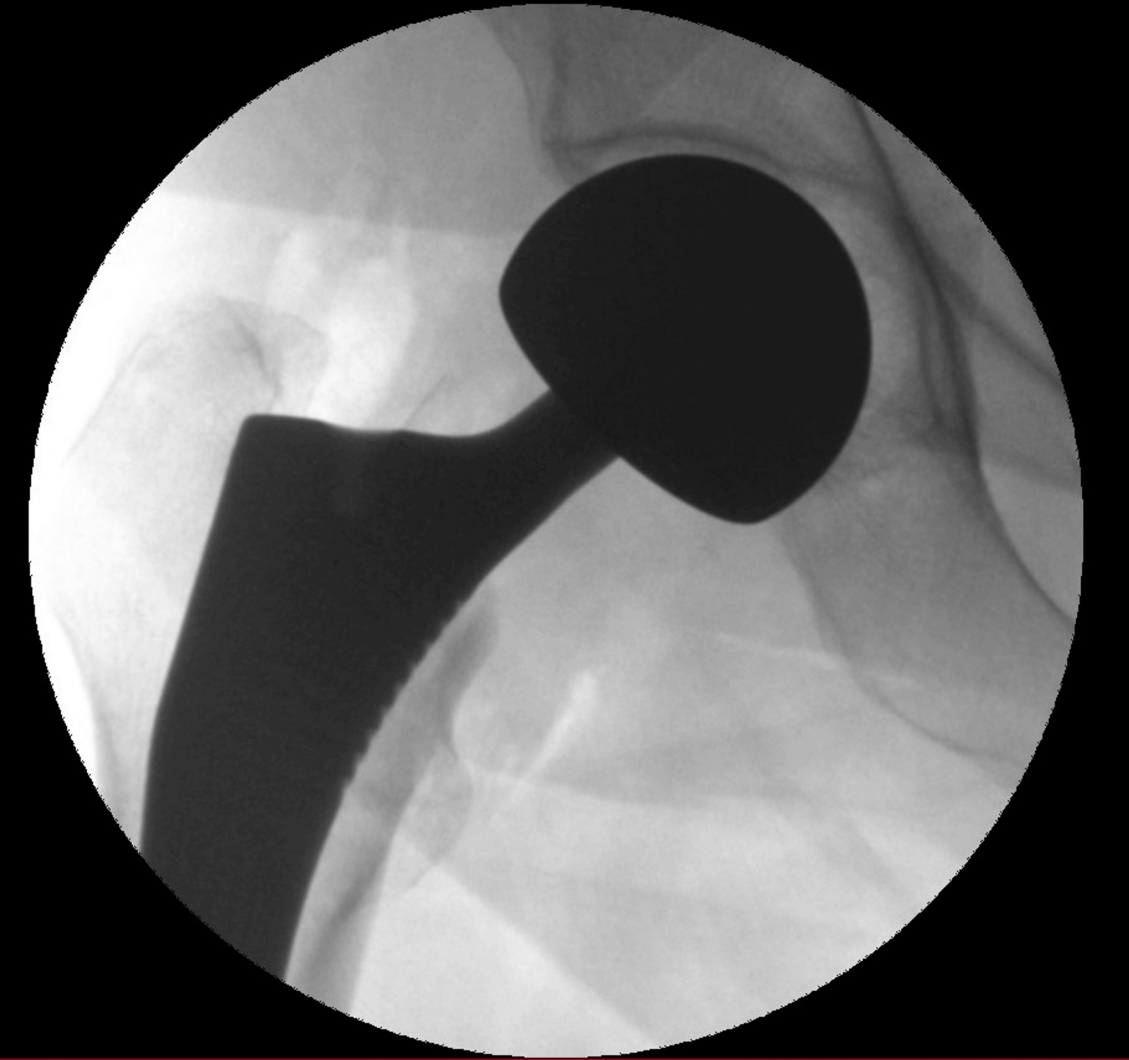
AP view of a bipolar uncemented hemiarthroplasty of the right hip for a basicervical femoral neck fracture AP: Anteroposterior

She also explained having episodes of productive cough and fever five days ago, treated with a three-day course of oral antibiotics (Azithromycin). She had no active respiratory symptoms. On presentation, she was vitally stable, afebrile, and oriented to name, place, and time. She complained of pain around her right hip region. Her right lower limb was externally rotated and marginally shortened. Distal pulses were palpable, and the toe capillary refill time was around two seconds. Her neurovascular status was intact in both lower limbs. The patient was able to ambulate independently prior to injury. She was admitted and prepared for hip arthroplasty with the aim of restoring her function and activity level. Her routine preoperative investigations revealed basic labs within normal limits (Table 1), including prothrombin time and international normalized ratio (INR). However, ill-defined pneumonitis in the lower zone of the right lung, and band atelectatic changes in the bilateral lower lung zones were reported on her chest X-rays. Her C-reactive protein (CRP) had a negligibly increased value. D-dimer was also increased at 1.48 (Normal < 0.5). Sputum cultures were also collected, and they were reported back negative. A nasal polymerase chain reaction (PCR) swab came back positive for human rhinovirus infection. A computed tomography pulmonary angiography (CTPA) scan was ordered, given her increased D-dimer, to rule out pulmonary embolism. The CT scan of the chest was reported as normal. Her surgery was delayed for some time until surgical clearance could be obtained from the pulmonology team. Eleven days after admission, the patient was taken to the operation theater for bipolar uncemented hemiarthroplasty of the right hip (Figure 2). The patient was positioned supine on a standard radiolucent table and received both general anesthesia and regional anesthesia via a pericapsular nerve group (PENG) block. A modified Watson-Jones approach was utilized for the right hip. The surgery proceeded smoothly without any intraoperative complications.

**Table 1:** Preoperative laboratory test results eGFR: estimated glomerular filtration rate

Test	Result	Normal range
White cell count	9.6	3.6 - 11.0 uL
Red cell count	3.58	3.80 - 4.80 uL
Hemoglobin	12.0	12.0 - 15.0 g/dL
Hematocrit	35.2	36 - 46 %
Platelet count	267	150 - 450 uL
Prothrombin time	13.9	11 -14 seconds
INR	1.11	0.8 - 1.11
APTT	37.9	28 - 41 seconds
Sodium	137	136 - 145 mmol/L
Potassium	4.8	3.3 - 4.8 mmol/L
Urea	62	12 - 40 mg/dL
Creatinine	1.13	0.50 - 0.90 mg/dL
eGFR	59.6	>60 ml/min/1.73m(2)
Procalcitonin	0.09	< 0.05 ng/mL
C-reactive protein	8.4	< 5.0 mg/L
D-dimer	1.48	< 0.5 ug/ml
HbA1C	5.3	< 5.7%

In the recovery area, an early postoperative assessment conducted a few minutes after surgery revealed a delayed capillary refill time of approximately 4 seconds in the right foot. Doppler signals for the dorsalis pedis and posterior tibial arteries were absent, and the right foot felt slightly cooler than the left, despite the patient being vitally stable. A pulse oximeter placed on the toe of the right foot showed oxygen saturation ranging from 88% to 98%.

**Figure 3:**
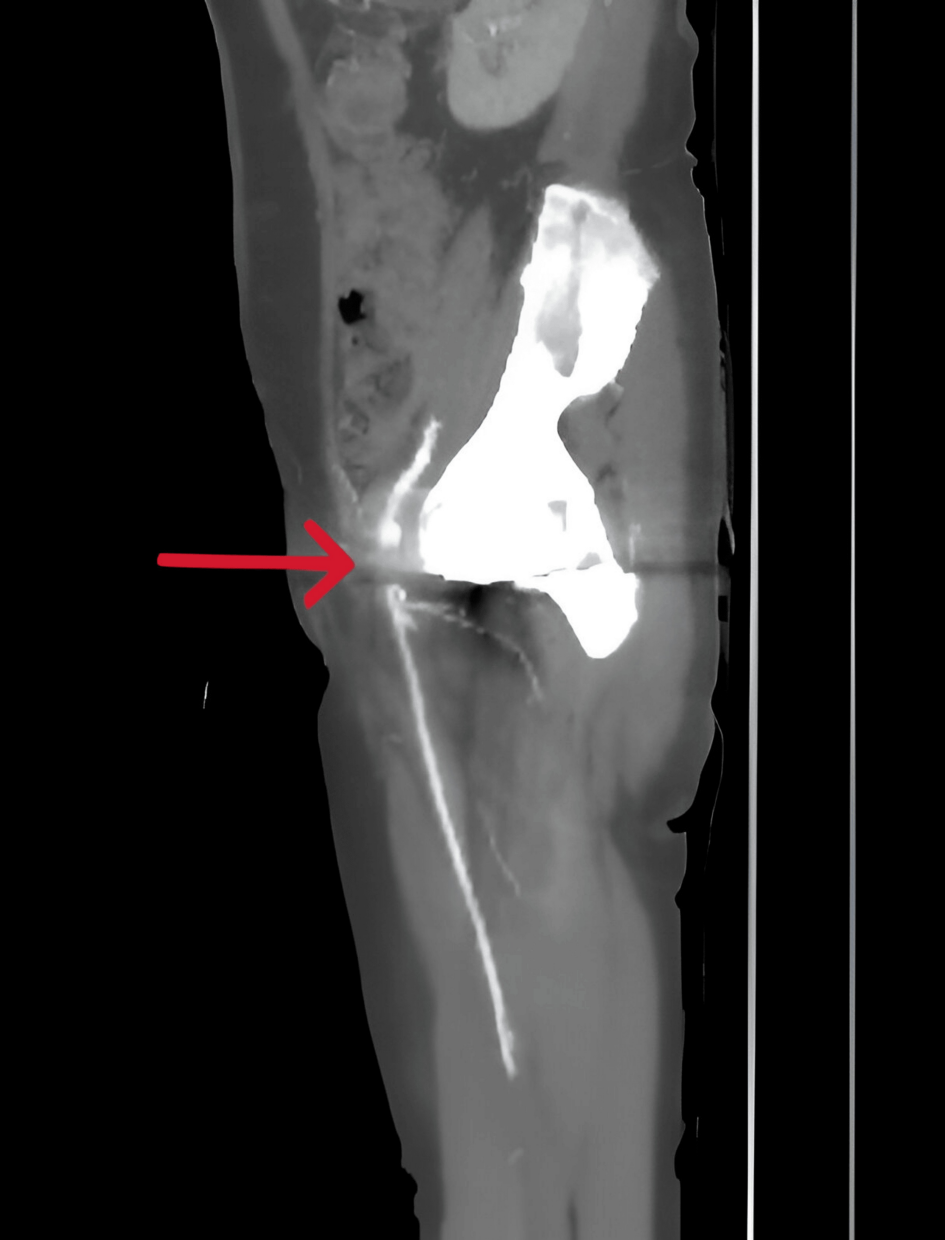
CT angiography of a right lower limb, which shows a non-opacification of a short segment (approximately 2 cm) of the right common femoral artery near its bifurcation (red arrow)

An urgent vascular consultation was requested. Upon evaluation, the vascular surgeon confirmed absent distal pulses via Doppler, while femoral and popliteal pulses were present but weak. The vascular surgeon ordered an urgent CT angiography of the right lower limb, which reported a non-opacification of a short segment (approximately 2 cm) of the right common femoral artery near the bifurcation (Figure 3).

This finding was possibly due to a metallic artifact from the hip prosthesis, but arterial occlusion could not be ruled out. The distal arteries were well opacified. The patient received a single dose of 40 mg of enoxaparin (Clexane).

The following day, the vascular surgery team performed an exploration in the operating room. Intraoperative findings included a focal injury to the common femoral artery and a dissected intimal plaque extending to the femoral bifurcation. An endarterectomy was performed to remove the plaque, the intima was repaired using 7/0 Prolene, and the artery was closed with a venous patch.

**Figure 4:**
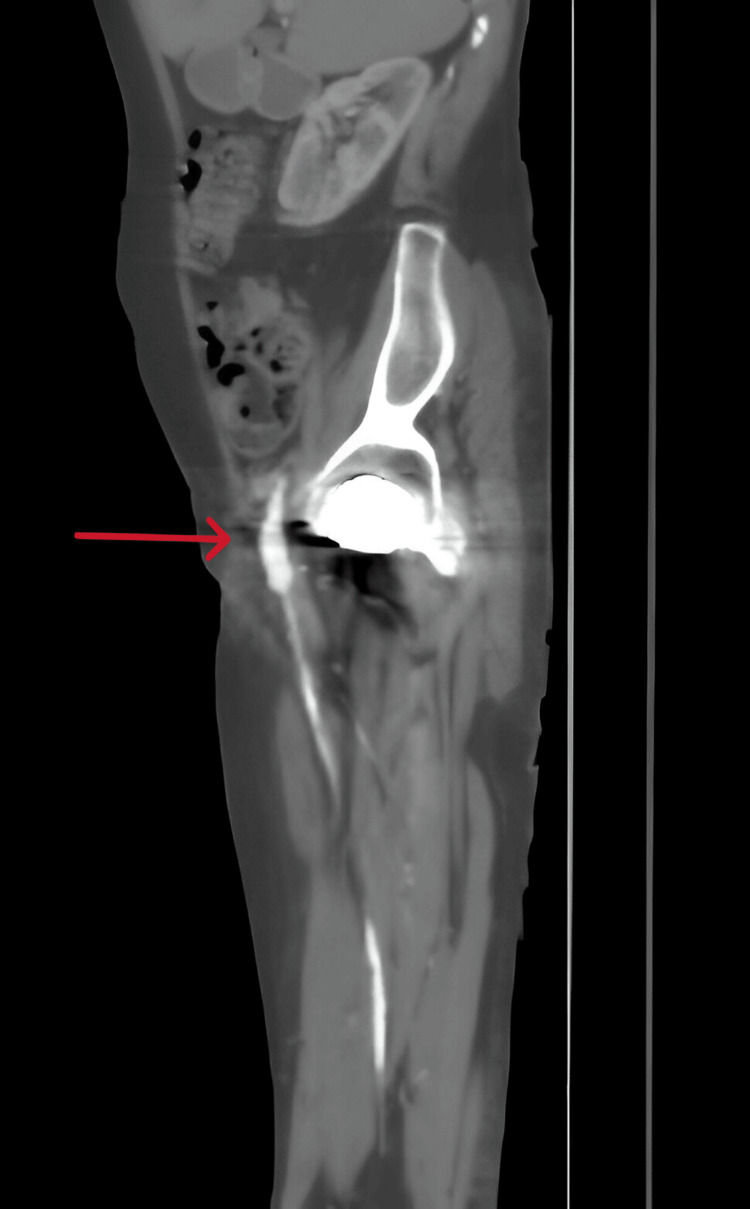
A follow-up CT scan showing resolution of the occlusion of the common femoral artery (red arrow).

Postoperatively, the distal pulses were clearly palpable, with triphasic Doppler signals confirmed (Figure 4).

The patient was monitored in the ward for several days and discharged after achieving full weight-bearing mobilization with a walker, assisted by physiotherapists. At discharge, the patient was prescribed anticoagulation therapy and advised on smoking cessation.

## Discussion

There is a minimal role for conservative management for femur neck fractures; it is considered in high-risk patients and critically ill patients and is very rarely implemented [9]. The surgical options are osteosynthesis (surgical fixation of the fracture), resection arthroplasty (excision of the femoral head and neck), or joint replacement surgery. Due to the bone quality and the risk of avascular necrosis of the femoral head, especially in the elderly population, surgical fixation may not be an effective option, and it has a high risk of failure and conversion to joint replacement surgery. Resection arthroplasty is one of the last resorts in seriously ill and medically unfit patients to relieve their pain; however, it has high re-operation and mortality rates and low functional outcomes [10]. The treatment of choice in the elderly population is joint replacement surgery, either partial (hemiarthroplasty) or total (total hip arthroplasty (THA)), depending on some patient factors, including age, quality of the bone, and the pre-injury function of the patient. Hemiarthroplasty (HA) has the advantages of early mobilization, excellent pain relief, and good return to function in the long term [11]. THA has a higher risk of dislocation in the first four years after surgery; afterwards, there is no difference compared to HA. Due to this difference, most of the less mobile elderly patients receive an HA for femur neck fractures; however, each case is reviewed individually for the best decision on the joint replacement modality. Mortality and infection rates are the same in both HA and THA [12]. A research study done in the United Kingdom (UK) in 2023 described that over 100,000 hip replacements are being done annually in the UK, with a low 10-year failure rate of only 4.56% [13]. The numbers are even higher in the United States, reaching over 2.8 million cases of hip and knee replacements annually, according to the American Joint Replacement Registry in 2022 [14].

We found two documented cases of common femoral artery intimal injury and appositional thrombosis reported in the literature [7]. The proposed mechanism of injury of the common femoral artery was theorized to be direct pressure from the Hohmann retractors used to expose the hip joint causing a mechanical strain on the atherosclerotic artery, causing an intimal tear and rupture of the atherosclerotic plaque and subsequent arterial thrombosis [5,8]. This mechanism of injury fits into our case, as there was no excessive bleeding intraoperatively, making arterial laceration unlikely. Our patient’s age, long-standing history of hypertension, and heavy smoking make them highly susceptible to atherosclerosis.

Intimal tears can also occur secondary to excessive traction of an atherosclerotic femoral artery, leading to intimal tears and thrombus formation. This can occur during traction for reduction and dislocation of the hip joint intraoperatively.

Ideally, thrombolysis should be performed within four to six hours of onset [15]. However, determining the exact timing of thrombosis is often difficult. In general, surgical management is typically successful if intervention occurs within 24 hours. In our case, the vascular surgery team operated on the patient the following day, still within 24 hours, achieving excellent limb perfusion.

Another potential cause of common femoral artery intimal damage is the heat generated during the polymerization of a cemented implant or from the use of cautery during dissection. However, heat from cementing does not apply here since our HA implant was uncemented. And while we consistently exercise caution during dissection near critical anatomical structures, iatrogenic injury from the cautery remains a possibility.

An inexperienced assistant surgeon could injure the important neurovascular structures while inserting some instruments like Hohmann retractors. This is not the case in our surgery, as in our hospital, the primary surgeon is the one who applies the Hohmann retractors or Langenbeck retractors and hands them to the assistant surgeons.

Difficult reduction of the hip joint after inserting the femoral prosthesis in the case of a tight hip might be a reason that causes vascular injuries similar to our presented case. It is worth keeping this in mind while struggling to reduce a tight hip and to consider reviewing the prosthesis size in a way that will ensure proper sizing of the prosthesis and avoid compromise of hip joint stability.

## Conclusions

Surgeons must always be mindful of the close proximity of the common femoral artery to the femoral head and acetabulum, as they are separated only by the joint capsule and the psoas muscle. When placing Hohmann retractors, surgeons should position them as close to the bone as possible, ensuring no soft tissue lies between the retractors and the bone. Excessive retraction over the acetabular rim should be avoided to reduce the risk of injury to the femoral vessels and nerves.

Although arterial thrombosis is a rare complication following hip replacement surgery, it is critical to assess the neurovascular status both preoperatively and postoperatively. Early recognition and prompt management of limb ischemia are essential to preserving the limb. Unfortunately, delayed diagnosis can necessitate measures as drastic as above-knee amputation in some cases, which remains an unavoidable outcome.
